# Job satisfaction in midwives and its association with organisational and psychosocial factors at work: a nation-wide, cross-sectional study

**DOI:** 10.1186/s12913-022-07852-3

**Published:** 2022-04-02

**Authors:** Malin Hansson, Anna Dencker, Ingela Lundgren, Ing-Marie Carlsson, Monica Eriksson, Gunnel Hensing

**Affiliations:** 1 Institute of Health and Care Sciences, Sahlgrenska Academy, University of Gothenburg, P O Box 457, 405 30 Gothenburg, SE Sweden; 2 Department of Health and Nursing, School of Health and Welfare, Halmstad University, Halmstad, Sweden; 3 Department of Health Sciences, University West, Trollhättan, Sweden; 4 School of Public Health and Community Medicine, Institute of Medicine, the Sahlgrenska Academy, University of Gothenburg, Gothenburg, Sweden

**Keywords:** Work satisfaction, Work environment, Midwifery, Professional autonomy, Salutogenesis, COPSOQ III

## Abstract

**Background:**

Midwives report a challenging work environment globally, with high levels of burnout, insufficient work resources and low job satisfaction. The primary objective of this study was to identify factors in the organisational and psychosocial work environment associated with midwives’ job satisfaction. A secondary objective was to identify differences in how midwives assess the organisational and psychosocial work environment compared to Swedish benchmarks.

**Methods:**

This nation-wide, cross-sectional web survey study analysed midwives’ assessment of their organisational and psychosocial work environment using the COPSOQ III instrument. A multivariable, bi-directional, stepwise linear regression was used to identify association with job satisfaction (*N* = 1747, 99.6% women). A conventional minimal important score difference (MID ± 5 as a noticeable difference with clinical importance) were used to compare midwives’ results with Swedish benchmarks.

**Results:**

A multivariable regression model with 13 scales explained the variance in job satisfaction (*R*^*2*^ = .65). Five scales, possibilities for development, quality of work, role conflict, burnout and recognition, explained most of the variance in midwives’ job satisfaction (*R*^*2*^ = .63) and had *β* values ranging from .23 to .10. Midwives had adverse MID compared to Swedish benchmarks with higher difference in mean values regarding quantitative demands (8.3), work pace (6.0) emotional demand (20.6), role conflicts (7.9) and burnout (8.3). In addition, lower organisational justice (-6.4), self-rated health (-8.8), influence (-13.2) and recognition at work (-5.8). However, variation and meaning of work showed a beneficial difference in mean values with 7.9 and 13.7 respectively.

**Conclusions:**

Midwives reported high levels of meaningfulness in their work, and meaningfulness was associated with job satisfaction. However, midwives also reported adversely high demands and a lack of influence and recognition at work and in addition, high role conflict and burnout compared to Swedish benchmarks. The lack of organisational resources are modifiable factors that can be taken into account when structural changes are made regarding organisation of care, management and resource allocation. Midwives are necessary to a high quality sexual, reproductive and perinatal health care. Future studies are needed to investigate if job satisfaction can be improved through professional recognition and development, and if this can reduce turnover in midwives.

## Background

Given the importance of midwives in all aspects of sexual, reproductive, maternal, newborn and adolescent health care, improvements in the work environment have been called for by the UNFPA as a way to recruit and retain midwives in the occupation [1]. Midwifery work is complex, including organisational and clinical demands, but the work is also rewarding and is a source of professional pride [2–5]. Low job satisfaction in midwives has previously been found to lead to burnout [6–8] and higher intention to leave [9]. An integrated review showed that high job satisfaction increased intention to stay in the midwifery profession [10]. Midwives in the Nordic countries have an autonomous professional responsibility and handle uncomplicated pregnancies and births independently [11] in a context where almost all births are carried out at obstetric-led hospitals [12]. This autonomy could be expected to improve job satisfaction, but few studies exist on midwives’ job satisfaction in the Nordic countries.

The authors’ previous qualitative studies conducted in Swedish labour wards, showed that midwives experienced a strained work environment characterised by an assembly line principle where role conflicts were present. Role conflicts emerged between midwives and obstetricians related to the principles of midwifery (births as normal events) and medicine (births as events in need of specialised medical support). These overarching different principles led to role conflicts at work including issues such as leadership, decision-making and support to the birthing woman. This lack of clarity regarding professional roles in the team around the birthing women, made interprofessional collaboration more difficult [13, 14]. However, midwives were also found to experience psychosocial resources at work, suggesting that a salutogenic perspective is relevant in addition to the workplace strains [2, 14]. A health-promoting facilitative condition in midwives’ work environments was having the opportunity to work autonomously as a midwife with enabling organisational prerequisites [2]. Such prerequisites, according to the qualitative analysis, enabled midwives to develop a professional identity and supported grounded knowledge and professional courage. Professional courage was identified as enabling midwives to find a pathway within the different fields of work included in the professional domain [2]. In the present study the qualitative research findings was further explored through quantitative measurements of the organisational and psychosocial work environment.

To sum up, although midwives work in strained environments, their work is meaningful, and they experience professional pride. Few studies exist in the context of the obstetric-led health care system were midwives work with high autonomy, as in Sweden. Reviews on midwives’ work situation have indicated a lack of research focusing on beneficial factors such as job satisfaction [10, 15] and there is limited research on midwives’ work situation in Sweden. Thus, there is a knowledge gap in line with aim of this article.

The primary objective of this study was to identify factors in the organisational and psychosocial work environment associated with midwives’ job satisfaction. A secondary objective was to identify differences in how midwives assess the organisational and psychosocial work environment compared to Swedish benchmarks.

## Methods

### Setting and participants

The analysis used baseline data of Swedish midwives from a nation-wide, cross-sectional web survey that was conducted in 2020. The study population consists of midwives, who worked as midwives and were members of the Swedish Association of Midwives and the Swedish Association of Health Professionals, which organise the great majority of unionized midwives in Sweden. Exclusion criteria was not working as a midwife (e.g. pensioner, student, other work etc.).

Data was collected between 4^th^ of February and 20^th^ April, 2020. Individual links to the survey were generated by a data collection company and were sent out by the unions to the registered e-mail addresses of all midwives who met the eligibility criteria. The unions sent out three reminders to the participants during the data collection period. The invitation to participate was sent to 5076 midwives, of whom 2060 responded, giving a response rate of 41%. The full analysis set in this study is 1747 midwives for whom we have available outcome data, including all responders with > 50% reported data on included QOPSOC III scales. The data collection company generated the database used in the analysis.

### Variables

#### Independent variables

Independent variables associated with job satisfaction were measured by the third version of the COPSOQ III instrument, on scale level, since it is a comprehensive instrument developed to assess the organisational and psychosocial work environment [16]. The scales are structured over the following domains: demands at work, work organisation and job content, interpersonal relations and leadership, social capital and health and well-being. Each domain consists of one or several scales. The COPSOQ instrument has been found to have satisfactory psychometrical properties [17, 18]. Participants responded to the items on a five-point Likert scale, scored with 100, 75, 50, 25, and 0 points, respectively. Some items were scored in reverse. The structure and properties of the independent variables i.e. scales in the COPSOQ III instrument are presented in Table 1. The scale measurement was recorded as missing if > 50% of the item responses were missing.

**Table 1:** Description of independent variables in COPSOQ III for Swedish midwives

Domains	Scales/Items	Independent variables/ scales	Items	Scale Min–Max	Low/ High positive	Response options^a^	Cronbach’s Alpha
Demands at Work	3/8	Quantitative demands	3	0–100	L	1	.78
		Work pace	2	0–100	L	1 & 2	.80
		Emotional demands	3	0–100	L	1 & 2	.66
Work Organisation and Job Content	5/11	Influence at work	4	0–100	H	1	.71
		Possibilities for development	3	0–100	H	2	.69
		Variation in work	1	0–100	H	1	One Item
		Meaning of work	1	0–100	H	2	One Item
		Quality in work	2	0–100	H	2	.81
Interpersonal Relations and Leadership	8/20	Predictability	2	0–100	H	2	.65
		Role clarity	3	0–100	H	2	.74
		Role conflicts	3	0–100	L	2	.69
		Quality of leadership	3	0–100	H	2	.85
		Social support from manager	2	0–100	H	1	.89
		Social support from colleagues	2	0–100	H	1	.80
		Recognition	2	0–100	H	2	.68
		Sense of community	3	0–100	H	1	.77
Social Capital	3/7	Vertical trust, management	3	0–100	H	2	.79
		Horizontal trust, employees	1	0–100	H	2	One Item
		Organisational justice	3	0–100	H	2	.76
Health and well-being	3/7	Self-rated health	1	0–100	H	3	One Item
		Stress	3	0–100	L	4	.85
		Burnout	3	0–100	L	4	.87
Sum	22/53						

^a^Explanation and values for the response options (each scale is scored in the direction indicated by the question):

1 = Always (100); 2 = Often (75); 3 = Sometimes (50); 4 = Seldom (25); 5 = Never/hardly ever (0)

2: To a very large extent (100), To a large extent (75), Somewhat (50), To a small extent (25), To a very small extent (0)

3: Excellent (100), Very good (75), Good (50), Fair (25), Poor (0)

4: All the time (100), A large part of the time (75), Part of the time (50), A small part of the time (25), Not at all (0)

Internal consistency of the COPSOQ III scales in this study was examined with Cronbach’s alpha with coefficients ranging from 0.65 to 0.89.

#### Dependent variable

The dependent variable, job satisfaction, was measured with a four-item scale from the COPSOQ III. The four questions about job satisfaction were as follows: Regarding your work in general, how pleased are you with (i) your work prospects? (ii) the physical working conditions? (iii) the way your abilities are used? (iv) your job as a whole everything taken into consideration?. Participants responded to these items on a five-point Likert scale ranging from Very satisfied (100), Satisfied (75), Neither/Nor (50), Unsatisfied (25), Very unsatisfied (0). The internal consistency of the dependent variable job satisfaction was found to be good with Cronbach’s alpha coefficient 0.82.

#### Potential confounders

Previous research on midwives’ job satisfaction and work environment has identified that age and work experience were associated with both the work environmental factors and job satisfaction [9, 19–21]. Thus, age and work experience were adjusted for in the regression analysis. The questions were (i) What is your age? (ii) How many years have you worked as a midwife?

#### Differences in assessment of the work environment between the midwives and Swedish benchmarks

The Swedish benchmarks for COPSOQ III scales were established by Berthelsen et al. for an adult working population in Sweden by including a random sample of 2847 and a convenience sample of 1818 gainfully employed people in Sweden, aged 25–65 years [17]. In studies with a large number of participants, it can be difficult to assess if a statistically significant result is of practical importance. Pejtersen et al. suggested a conventional minimal important score difference (MID) of ± 5 as a noticeable difference with clinical importance [22]. The pre-defined MID between our study sample of midwives and the Swedish benchmarks for the COPSOQ III scales were analysed to assess the organisational and psychosocial work environment for midwives [17].

### Statistical analysis

Univariable linear regression analyses were performed with job satisfaction as a dependent variable. The assumption of normal distribution and homoscedasticity of residuals as well as the assumption of linear functional form was examined by diagnostic plots of the COPSOQ III scales and potential confounders. Multicollinearity was rejected for all variables with variance inflation factors between 1 and 2.5 [23]. The independent variables were first analysed separately in univariable analyses and were thereafter adjusted for age and amount of work experience as a midwife. Age and work experience were strongly correlated *r* = 0.87, and work experience had a better degree of explanation. Therefore, only work experience was adjusted for in this and further analyses. In the univariable regression analyses, the full analysis set varied from 1754 to 1911 in the different COPSOQ scales.

In accordance with the predefined statistical analysis plan all the independent variables that were significant (*p* < 0.05) were included in a multivariable regression model, using bi-directional stepwise regression, hence, forward selection and backward elimination. We report beta estimates with 95% CI, associated *p*-value and amount of explained variance R^2^ from the univariable and multivariable regression models in Table 3. The full analysis set in the multivariable regression model was 1747 midwives after excluding 313 individuals with missing values.

In assessing midwives’ work environment, scales were computed as the mean of items, and standard deviation was analysed for each COPSOQ III scale. One sample t-tests were conducted to analyse the difference between the midwives’ means and the Swedish benchmarks as well as to analyse whether there was at least a predefined MID between the groups. All tests were two-sided, and an alpha level of 0.05 was applied. Adjustment of multiple inference was made applying Bonferroni-Holm step-down procedure. The results are presented in Table 4. In this analysis, the full analysis set varied from 1754 to 1911 for the different COPSOQ scales.

## Results

### Participants

Participant characteristics are shown in Table 2. The mean age was 48 years, 82% were married or lived in a stable relationship and 56% had children under 18 years of age living at home. The mean work experience was 16 years, and the mean amount of time at the current workplace was eight years. Only 52% worked full time, but 95% had permanent employment. The places of work varied, and included labour ward (44%), maternity care (32%), postnatal care (29%), gynaecology (11%) and youth clinic (8%). ‘Other’ could indicate a combination of these workplaces or, for, example an abortion clinic or breastfeeding clinic.

**Table 2:** Participant characteristics of Swedish midwives (*N* = 1747)

	*N*^a^	%	
*Gender*			
Female	1691	99.6	
Male	3	0.2	
Other	3	0.2	
*Civil status*			
Living alone	219	13	
Married/living in a stable relationship	1392	82	
Other living arrangements	86	5	
*Children under 18 years living at home*			
Yes	951	56	
No	746	44	
*Where do you work*^b^			
Labour ward	756	44	
Postnatal care	486	29	
Maternity care	550	32	
Gynaecology	189	11	
Youth clinic	142	8	
Other country	9	0.5	
Other (e.g., breastfeeding/abortion/antenatal clinic)	315	19	
*Main employment*			
Permanent employment	1618	95	
Temporary employment	64	3.7	
Self-employed	8	0.5	
Other	7	0.4	
*Employment status*			
Full-time	877	52	
Part-time	809	48	
Not employed	11	0.6	
	*mean*	*SD*	*Range*
*Age*	48	10.44	25–70
*Work experience as a midwife*	16	11.17	1–47
*Years at current workplace*	8	8.79	1–48

^a^There were available data on participant characteristics in 1697 participant due to that these variables were at the end of the extensive survey and therefore had missing values

^b^Some participants had multiple workplaces. The percentage given is in relation to the number of answering participants on the question *N* = 1697

### Multivariable stepwise linear regression analysis

In the univariable regression analysis, all independent variable scales were significant, and they were still significant after controlling for the confounder ‘amount of work experience as a midwife. Thus, all scales were included in the multivariable regression model, using bi-directional stepwise regression (Table 3).

**Table 3:** Univariable and multivariable regression with job satisfaction as a dependent variable, Swedish midwives (*N* = 1747)

***Scales***	***Univariable regressions***^**a**^					***Multivariable bi-directional stepwise regression model***			
	***β***	***(95% CI)***	***Sig***	***R***^***2***^	***N***	***β***	***(95% CI)***	***Sig***	
Possibilities for development	.62	.57, .66	< .001	.296	1909	.23	.19, .27	< .001	***R***^***2***^** = *.65*** *N* = *1747*
Quality of work	.63	.60, .67	< .001	.393	1911	.18	.14, .22	< .001	
Role conflicts	-.54	-.58, -.50	< .001	.283	1910	-.11	-.15, -.07	< .001	
Burnout	-.48	-.51, -.44	< .001	.305	1754	-.10	-.14, -.07	< .001	
Recognition	.53	.50, .56	< .001	.399	1910	.10	.07, .14	< .001	
Influence at work	.56	.52, .60	< .001	.256	1908	.09	.06, .13	< .001	
Vertical trust, management	.58	.55, .62	< .001	.353	1791	.09	.05, .13	< .001	
Sense of community	.59	.54, .64	< .001	.213	1907	.08	.04, .13	< .001	
Emotional demands	-.42	-.47, -.37	< .001	.119	1909	-.07	-.11, -.03	.001	
Meaning of work	.37	.31, .43	< .001	.072	1907	.07	.03, .11	.002	
Quality of leadership	.46	.43, .50	< .001	.296	1785	.06	.03, .10	< .001	
Variation of work	.26	.21, .30	< .001	.070	1907	.04	.01, .07	.012	
Self-rated health	.36	.33, .40	< .001	.206	1754	.04	.01, .07	.005	
Organisational justice	.62	.59, .66	< .001	.384	1791				
Predictability	.61	.57, .65	< .001	.330	1910				
Social support from manager	.39	.36, .42	< .001	.266	1791				
Stress	-.44	-.47, -41	< .001	.264	1754				
Role clarity	.57	.52, .62	.000	217	1910				
Social support from colleagues	.43	.38, .47	< .001	.165	1907				
Work pace	-.37	-.41, -.33	< .001	.146	1907				
Horizontal trust, employees	.38	.33, .43	< .001	.115	1785				
Quantitative demands	-.35	-.39, -.30	< .001	.114	1908				
***Confounder***									
Work experience as a midwife	.32	.24, .40	< .001	.034	1695				

*β* estimates with 95% confidence interval (CI), associated *p*-value with an alpha level of .05, *R*^*2*^ proportion of the variance explained by the model

All *p*-values (in both univariable and multivariable analyses) remained statistically significant after applying Bonferroni-Holm adjustment for multiple comparisons allowing total significance level to be 0.05

^a^Adjusted for work experience

Thirteen scales were included in the multivariable regression model (Table 3) with an explanation of the variance in job satisfaction *R*^*2*^= 0.65. The scales explained 65% of how the midwives rated their job satisfaction. All included scales made a unique contribution to the explained variance of job satisfaction with significant values ranging from < 0.001 to 0.012 [23]. However, the first five scales possibilities for development, quality of work, role conflicts, burnout and recognition, explained most of the variance in midwives’ job satisfaction (*R*^*2*^ = 0.63) and had *β* values ranging from 0.23 to 0.10.

### Differences in assessment of the work environment between the midwives and the Swedish reference values

When analysing the difference in mean values between the midwives’ results and Swedish benchmarks for COPSOQ III, the difference reached a predefined MID between the groups in eleven of the scales (Table 4). The midwives reported adverse MID (marked with ** in Table 4) compared with the reference population in terms of higher quantitative and emotional demands, faster work pace, more role conflicts and more burnout. Midwives also reported lower influence at work, recognition, organisational justice and self-rated health. However, the scales for variety and meaningfulness of work beneficially differed with a higher MID than the Swedish benchmarks (marked with *** in Table 4).

**Table 4:** Midwives in Sweden (*N* = 1754–1911); mean scores and standard deviation (SD) of COPSOQ III scales compared to Swedish reference values

COPSOQ III scales	High/ Low Levels Positive	Midwives in Sweden 2020 Mean (SD)	Swedish bench-marks Mean	Difference in mean values* (95% CI)	*p*-value
Quantitative demands**	L	49.2 (18.6)	40.9	8.3 (7.5–9.1)	< .001
Work pace**	L	65.5 (20.0)	59.5	6.0 (5.1–6.9)	< .001
Emotional demands**	L	67.4 (16.0)	46.8	20.6 (19.8–21.3)	< .001
Influence at work**	H	37.0 (17.2)	50.2	-13.2 (-14.0– -12.5)	< .001
Possibilities for development	H	72.5 (16.9)	70.4	2.1 (1.4–2.9)	< .001
Variation of work***	H	75.9 (19.6)	68.0	7.9 (7.0–8.7)	< .001
Meaning of work***	H	92.0 (13.9)	78.3	13.7 (13.0–14.3)	< .001
Quality of work	H	64.0 (19.0)	68.2	-4.2 (-5.0– -3.3)	< .001
Predictability	H	59.8 (18.1)	60.2	-0.4 (-1.2–0.4)	.298
Role clarity	H	77.2 (15.7)	78.1	-0.9 (-1.6 – -0.2)	.011
Role conflicts**	L	50.1 (18.9)	42.2	7.9 (7.1–8.8)	< .001
Quality of leadership	H	51.4 (22.7)	54.1	-2.7 (-3.7 – -1.6)	< .001
Social support from manager	H	70.4 (25.6)	75.3	-4.9 (-6.2 – -3.8)	< .001
Social support from colleagues	H	79.2 (18.4)	80.2	-1.0 (-1.9 – -0.2)	.012
Recognition**	H	59.8 (23.0)	65.6	-5.8 (-6.9 – -4.8)	< .001
Sense of community	H	78.7 (15.0)	79.9	-1.2 (-1.9 – -0.6)	< .001
Job satisfaction	H	64.2 (19.1)	64.4	-0.2 (-1.1–0.6)	.633
Vertical trust, management	H	64.4 (19.6)	69.3	-4.9 (-5.8 – -4.0)	< .001
Horizontal trust, employees	H	74.7 (17.3)	71.3	3.4 (2.6–4.2)	< .001
Organisational justice**	H	53.3 (19.1)	59.7	-6.4 (-7.2 – -5.5)	< .001
Self-rated health**	H	52.5 (24.0)	61.3	-8.8 (-10 – -7.7)	< .001
Stress	L	40.2 (22.5)	36.0	4.2 (3.1–5.2)	< .001
Burnout**	L	44.5 (22.3)	36.2	8.3 (7.3–9.4)	< .001

^*^Pejtersen et al. suggested a conventional minimal important score difference (MID) of ± 5 as a noticeable difference with clinical importance for the employee [22]

^**^ Adverse MID from Swedish reference value [17, 22]

^***^ Beneficial MID from Swedish reference value [17, 22]

The largest differences in mean values were seen in the scales emotional demands, meaningfulness of work and influence at work. Midwives had a difference in mean values of 20.6 higher emotional demands compared to the Swedish benchmarks and –13.2 for influence at work, although the midwives also reported 13.7 higher meaningfulness of work. In other words, the midwives reported higher emotional demands and lower influence at work, but on the other hand, they also reported a higher level of meaningfulness in their work.

## Discussion

In this study, the aim was to identify factors associated with job satisfaction in midwives and to compare how midwives assessed their work environment with Swedish reference data. In the final model, thirteen scales were identified that explained 65% of the variance in how midwives scored on job satisfaction. These scales represent different aspects of the organisational and psychosocial work environment. When comparing midwives’ assessment of their work environment with Swedish benchmarks, we found that midwives reported significantly more adverse values for work pace, role conflicts, burnout, quantitative and emotional demands, influence, recognition, organisational justice and self-rated health. However, midwives beneficially differed from the reference data with higher values for meaningfulness and variety of work.

### Beneficial work environmental factors

The regression analyses revealed beneficial factors in the organisational and psychosocial work environment with variety and meaningfulness of work that were associated with midwives’ job satisfaction. Only these two scales had beneficial MID with higher values than the Swedish benchmarks. These findings are in line with our previous qualitative research, indicating that midwives’ work is highly varied and enables midwives to autonomously develop professional knowledge and skills with support from relevant organisational prerequisites [2]. The same applies to meaningfulness of work, where midwives’ relationships with pregnant and birthing woman and their partners gives them a feeling of being professionally useful. This is in line with Bloxsome et al. [10, 24], who emphasise the importance of making a difference and being of use. Other beneficial factors that were associated with job satisfaction in midwives included being able to influence the work being done and being able to provide high quality care in a context with prerequisites for professional development and recognition. These results correspond with an integrated review of midwives’ job satisfaction and intention to stay in the profession [10].

### Adverse work environmental factors

Factors with an adverse association with job satisfaction were high levels of burnout, role conflicts and emotional demands. In addition, we found that midwives adversely differed from the reference population in terms of work pace, quantitative and emotional demands, role conflicts and burnout as well as reporting lower levels of influence, recognition, organisational justice and self-rated health. Thus, our study found that midwives work in an organisational and psychosocial work environment characterised by high demands and low control, which is supported by previous research [2, 13, 14, 25–27]. In this study, emotional demands, in particular, adversely differed from the Swedish benchmarks with a difference in mean values of 20.6 higher emotional demands of midwives. The high emotional demands in midwifery have previously been described [28, 29] and the midwifery profession is known to be inherently emotional demanding. Our results are in line with previous research about midwives’ work environment, which has consistently found that midwives have a demanding work situation [2, 13, 14, 25, 27, 30]. In addition, midwives have been found to experience high levels of work-related stress [25, 27, 30], burnout [25–27, 31–33], poor organisational climate, insufficient work resources and under-staffing [26, 30]. A qualitative study of midwives’ emotional work found that conflicting ideologies in the organisation can be a source of additional emotional demands and ethical stress that can aggravate the work situation further due to competing ethical standpoints [34].

Role conflict and recognition at work were included in the multivariable model and were together with influence at work and organisational justice, scales that adversely differed from the Swedish benchmarks. These results are in line with two reviews of midwives’ work environments [10, 19], which described the importance for midwives of having influence at work and being able to practice midwifery autonomously without role conflict. Receiving recognition and working in a just organisation were shown to be the main determinants of job satisfaction according to Papoutsis et al. [35]. Similar results were obtained by Dixon et al. [29], who found that midwives’ emotional well-being was affected by professional recognition. Consequently, it is worrying that midwives in Sweden report low influence at work, high role conflicts, low recognition and low organisational justice, which are fundamental components of the organisational and psychosocial work environment. It is equally concerning that midwives’ assess their self-rated health significantly lower than does the reference population. Poor self-rated health has been shown to be an independent risk factor for both morbidity and mortality [36]. It is notable that midwives’ job satisfaction is associated with burnout and that gainfully employed midwives scored significantly higher than the Swedish benchmarks on the burnout scale. These results are in line with previous research on burnout in midwives [6, 27, 33, 37–39].

### The results in relation to the salutogenic theory and professional autonomy in midwifery

The exploratory approach taking into account multiple factors was informative since both positive and negative factors in the midwives’ organisational and psychosocial work environment were identified. Particularly interesting was the identification of beneficial factors in midwives’ work environment, which supports the importance of a salutogenic perspective on the organisational and psychosocial work environment in addition to the more traditional risk factor focus.

Researchers in salutogenic theory argue that resources and stressors in the work situation can be perceived as both positive and negative. Thus, a specific factor in the work environment cannot necessarily be designated as a stressor but, rather, the outcome of the factor depends on the work context and individual characteristics [40, 41]. This can be interpreted as an opportunity for employers to support and facilitate consistency and balance between underload and overload. The salutogenic theory also emphasises the importance of participating in decision-making [41, 42]. Another assumption in the salutogenic theory is that high demands at work can be balanced with a strong individual sense of meaningfulness and by the perception that work is comprehensible and manageable [41]. This sense of coherence generates the ability to use one’s resources to minimize the impact of the stressors. Thus, Antonovsky and Mittelmark mean that a sense of coherence can be seen as a personal resource that reduce work strain and lead to a perception of stressors as challenges rather than threats [43, 44].

The present results also indicate that the ability to influence one’s own work and provide high quality care was associated with job satisfaction. Previous research has found midwives’ professional identity and autonomy to be important in supporting a health-promoting work situation and job satisfaction [2, 3, 13, 19, 45]. Other associations with job satisfaction in this study were being recognised and respected in the professional scope of practice without role conflicts. A review of midwives’ job satisfaction obtained similar results, finding that job satisfaction was negatively affected by insufficient time for professional activities, low autonomy and high demands [19]. This aligns well with salutogenic theory, which highlights that the ability to work autonomously can lead to increased meaningfulness and motivation and can also balance high demands [40].

In order to achieve a health-promoting workplace, it is important to strengthen the workplace’s health-promoting factors, but also to work preventively based on the risk factors that exist in the specific workplace. A salutogenic assumption is that each individual, workplace and organisation has resources that can be used to maintain and develop health and a sense of coherence [40]. However, the specific resources and stressors of the workplaces need to be identified, which this study has contributed to for the field of midwifery.

### Strengths and limitations

The main strengths of this study are its nation-wide sample of midwives and its focus on both positive and negative factors in the work environment of midwives. Another strength is the diversity of the participants; for example, midwives’ place of work varied, whereas previous research has generally focused on the work environment in labour wards or inpatient care. Another strength is that, besides investigating the demands in the workplace, this study focuses on the workplace characteristics that contribute to job satisfaction.

No causal assumptions or conclusions can be made based on this study due to the cross-sectional design. Selection bias cannot be ruled out, due to possible differences between the midwives who are members of unions and those who are not. Recruiting midwives through the unions was an efficient way to reach the greatest number of Swedish midwives and still have control over who was included. Due to General Data Protection Regulation, we had to invite the midwives trough the union’s membership register. The unions sent out the invitations. Unfortunately, due to General Data Protection Regulation, we do not have any data on the non-responders. However, the gender distribution in our study is in line with the national statistics of midwives in Sweden. A sampling bias could be another possible limitation as there may be differences between the midwives who completed the survey and those who did not. We consider the response rate of 41% to be acceptable and have not found any discrepancy in the distribution of midwives in our sample compared to public statistics on midwives.

Another conceivable limitation in this study is that there were available participant characteristics in 1697 participant (2,9% less participants than the full analysis set) due to that these variables were at the end of the extensive survey and therefore had missing values. We chose to include all participants for whom we had available outcome data and with > 50% reported answers on included QOPSOC III scales in the regression analysis, to make use all reported data.

We aimed to give an overall perspective of midwives’ organisational and psychosocial work environment and kept the adjustment variables to age and years of work experience. Future studies are needed on specific groups of midwives (e.g. maternity ward vs gynaecological ward, part-time vs full-time, leadership vs not leadership). Selection bias as a reason for found differences with the benchmark population is considered less likely since the distribution of age and gender of midwives is in line with the national statistics, and since the found differences are in line with findings from other studies and our qualitative studies.

Further longitudinal research is needed to identify predictors of job satisfaction for midwives in Sweden by following the work situation over time to enable causal assumptions.

## Conclusion and clinical implications

Midwives reported high levels of meaningfulness in their work, and meaningfulness was associated with job satisfaction. However, midwives also reported adversely high demands and a lack of influence and recognition at work and in addition, role conflicts and burnout compared to Swedish benchmarks. The lack of organisational resources are modifiable factors that can be taken in to account when structural changes are made regarding organisation of care, management and resource allocation. Midwives are necessary to a high quality sexual, reproductive and perinatal health care. Future studies are needed to investigate if job satisfaction can be improved through professional recognition and development, and if this can reduce turnover in midwives.

## Data Availability

The dataset generated and analysed during the current study is not publicly available as individual privacy could be compromised, but it is available from the corresponding author on reasonable request.

## Abbreviations

MID: Minimal Important score Difference
COPSOQ: The Copenhagen Psychosocial Questionnaire
